# Research on quantitative detection technology of raccoon‐derived ingredient adulteration in sausage products

**DOI:** 10.1002/fsn3.3976

**Published:** 2024-01-23

**Authors:** Hui Wang, Chen Chen, Mengying Xie, Yan Zhang, Boxu Chen, Yongyan Li, Wenshen Jia, Jia Chen, Wei Zhou

**Affiliations:** ^1^ Hebei Food Safety Key Laboratory, Key Laboratory of Special Food Supervision Technology for State Market Regulation, Hebei Engineering Research Center for Special Food Safety and Health Hebei Food Inspection and Research Institute Shijiazhuang China; ^2^ Institute of Quality Standard and Testing Technology Beijing Academy of Agriculture and Forestry Sciences Beijing China; ^3^ College of Chemical Technology Shijiazhuang University Shijiazhuang China

**Keywords:** droplet digital PCR, quantitative study, raccoon, sausage adulteration

## Abstract

This project presents a quantitative detection method to identify raccoon‐derived ingredient adulteration in sausage products. The specific copy gene of the raccoon was selected as the target gene. According to the specificity of its primer and probe, the quantitative detection method of raccoon microdrops by droplet digital PCR was established. In addition, the accuracy of the proposed method was verified by artificially mixed samples, and the applicability of this method was tested based on the commercially available products. The experimental results indicate that the raccoon mass (*M*) and raccoon‐extracted DNA concentration have a good linear relationship when the sample content is 5–100 mg, and there is also a significant linear relationship between DNA content and DNA copy number (C) with *R* ^2^ = .9982. Therefore, using DNA concentration as the median signal, the conversion equation between raw raccoon mass (*M*) and DNA copy number (C) could be obtained as follows: *M* = (C + 177.403)/16.954. The detection of artificially mixed samples and commercial samples shows that the method is accurate and suitable for quantitative adulteration detection of various sausage products in the market.

## Graphical abstract

This manuscript describes a laboratory method for quantitative detection of raccoon‐derived components in sausage. Using DNA concentration as the median signal to establish the equation between raccoon meat mass and DNA copy number. Besides, the applicability of this method was verified through artificial adulteration models and 50 different commercial sausage products.

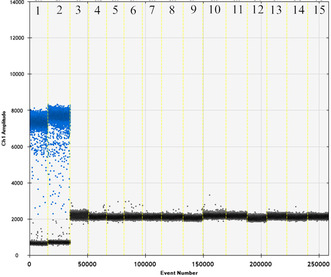

## 1 INTRODUCTION

Presently, there exists a large number of raccoon breeding facilities worldwide. The ultimate fate of the meat is uncertain subsequent to the extraction of fur, and the meat is not quarantined due to the fact that it is not regarded as a consumable food ingredient. Once these nonedible farmed meat sources are adulterated and mixed into the market, it would cause disorder in the consumer market and seriously damage the physical health of consumers. Several notable scandals, such as the Australian horse meat incident and the Wal‐Mart fox meat incident, serve as exemplars of such occurrences on a global scale (Tibola et al., 2018; O'Mahony, 2013). In addition, the recent incident of using mouse heads instead of duck necks in university canteens in Chinese schools also indicates that food adulteration is ubiquitous (Xianwei, 2022). Meat sausage is a processed food primarily made from poultry meat. After mincing and marinating the meat, it is filled into the casing and then processed through a series of processes to ultimately produce sausages (Ballin et al., 2009; de Oliveira et al., 2020). They are difficult to identify by appearance with the help of various edible flavors, which gives these nonedible meat sources adulterated in all kinds of meat sausage provides opportunities. However, there are still many shortcomings in the current food testing standards, and there is no standard testing method for special non‐edible meat sources. Establishing a quantitative, accurate, and effective method for detecting nonedible meat sources can therefore fill the gaps in global food testing standards research/surveillance.

At present, the identification of animal‐derived components identification mainly involves physical, chemical, immunological, and molecular biological methods (Liu et al., 2006; Tian et al., 2013; Zhang et al., 2022). Among these methods, ordinary PCR or fluorescent PCR techniques based on molecular biology have emerged as significant tools for detection (Ali et al., 2012; Wu et al., 2013). The detection of specific endogenous genes in animal‐derived ingredients by PCR technology, which is used to achieve adulteration identification, is a relatively complete adulteration technology. The sensitivity and accuracy of these technologies are relatively good technical tools. However, these technologies can only be used for qualitative determination to determine whether the sample contains certain animal‐derived ingredients, but cannot be used for quantitative detection to distinguish whether the adulterated ingredients in the problem sample are unintentionally contaminated or intentionally added, which creates certain obstacles to market law enforcement (Bharuthram et al., 2014; Cheng et al., 2020; Polanowski et al., 2012; Wang et al., 2022). Therefore, this paper employs droplet digital PCR (ddPCR) technology to quantitatively identify the adulteration of raccoon components.

Droplet digital PCR technology is a technique used for absolute nucleic acid quantitative detection. ddPCR microdrops the reaction system containing template DNA before the PCR reaction, disperses the template DNA molecules in about 20,000 microdrops, independently carries out PCR amplification on each microdrop reaction unit, reads the fluorescence signal, and calculates the number of template DNA molecules added to the system according to Poisson distribution principle (Massanella et al., 2013). Because it does not need to establish a standard curve, it is highly sensitive and accurate for absolute quantification of the copy number of nucleic acid at low concentrations of nucleic acid content (Whale et al., 2013). Therefore, ddPCR technology is applied in multiple fields such as microbial detection (Blaya et al., 2016; Chen et al., 2017; Zhang et al., 2016), genetically modified food detection (Köppel et al., 2015), and animal‐ and plant‐derived adulteration detection (Cai et al., 2017).

In this paper, the relationship between raccoon mass and its DNA concentration was studied using raccoon lean meat as the sample for the first time, and DNA quantification was performed by ddPCR based on the specific primers of raccoon genes. DNA concentration was used as the median signal to establish the equation between raccoon meat mass and DNA copy number. Besides, the applicability of this method was verified through artificial adulteration models and 50 different commercial sausage products. Finally, a fast, accurate, and quantitative detection method for raccoon meat adulteration in sausage products was established. This study not only fills the gap in the detection standards for non‐edible meat sources but also accurately quantifies adulterated raccoon meat, effectively distinguishing it from intentional adulteration or unintentional introduction during processing.

## 2 MATERIALS AND METHODS

### 2.1 Experimental materials

Raccoon and fox meat were obtained at Changli Fox and Raccoon Breeding Base (Qinhuangdao, China). Fresh lean meat samples of beef, sheep, duck, pork, and chicken were collected from local supermarkets (Shijiazhuang, China).

### 2.2 Reagents and laboratory apparatus

Primers and probes were synthesized by Shanghai Bioengineering Co., Ltd. Anhydrous ethanol was obtained from Beijing Luqiao Company. 2× ddPCR supermix for probes, droplet generation oil, and droplet reader oil were obtained from Bio‐Rad Company. GMO food DNA extraction kit was provided by Tiangen Company. The Electronic balance (ME204/02) was provided by Mettler Toledo International Co., Ltd.

### 2.3 Experimental method

#### 2.3.1 DNA extraction

Before DNA extraction, water in raw meat should be removed to avoid affecting the results of DNA quantitative detection. The fresh meat was ground by the meat grinder and then dried in the drying oven at 80°C for 72 h. After drying, the meat sample was cut in the sterilized mortar with liquid nitrogen, and then ultrafine powder was obtained, which was used as the experimental sample. After DNA extraction, the precipitate was air‐dried at room temperature and dissolved in 100 μL ddH_2_O (double‐distilled water). The concentration of extracted DNA was measured by Nanodrop 2000c spectrophotometer.

#### 2.3.2 Raccoon primer design

The raccoon‐based primer and probes were designed based on F2 gene sequence (GenBank: NW_0118846437.2) (Junan, 2017). The raccoon gene primer and probes sequences are depicted in Table 1.

**TABLE 1:** Raccoon gene primer and probe sequences.

Primer/probe	Sequence/labeling (5′⟶3′)
Raccoon F	TTGACTCCCAGGGCAGTCTCAG
Raccoon R	GAGCCTAATGTGCCCTCCATG
Raccoon Probe	FAM‐TAGCGCCACCTTCAGCCCAGGGC‐BHQ1

#### 2.3.3 ddPCR reaction system

Before performing the reaction analysis, the extracted DNA was diluted 30 times. The ddPCR program for the reaction denaturation was as follows: 4 μL of the template DNA, 1.2 μL of both upstream and downstream primers, 10 μL of ddPCR Supermix™ for probes (No dUTP), 0.4 μL of the probe, and 3.2 μL of sterile ddH_2_O which used as a blank control were utilized. The reaction system was mixed and transferred to the droplet‐generating card according to the instructions of ddPCR. Microdroplets were generated in the droplet generator and then amplified by PCR. The program for the amplification was carried out as follows: 95°C for 10 min, 40 cycles of 94°C for 30 s, 61°C for 1 min, 98°C for 10 min, and 4°C for temporary storage. Finally, microtiter plates were analyzed by the automatic microtiter plate reader. The results were calculated by quanta software after the signal acquisition.

#### 2.3.4 Primer and probe specificity

The specificity of primers and probes was evaluated by ddPCR reactions, including the DNA extracted from raccoon, fox, beef, sheep, chicken, pork, duck, and the negative control (water blank). The experimental reaction system and operating procedures are the same as described in Section 2.3.3.

#### 2.3.5 Establishment of the conversion formula between raccoon mass and copy number

##### Relationship between raccoon weight and extracted DNA concentration

Five to eighty microgram of raccoon samples was weighed and genomic DNA was extracted by the genomic DNA extraction kit. The DNA concentration of each sample was measured by the Nanodrop2000. Experiments were performed in triplicate. Then, the standard curve between the weight of the sample and extracted DNA concentration was established.

##### Relationship between DNA content and copy number of raccoon meat

The DNA concentration of extracted raccoon meat was diluted in a series of gradient dilution sequences, which were 5, 10, 20, 30, 40, 50, 60, 70, and 80 ng/μL, respectively. The ddPCR detection was performed, and each of the processes was repeated three times. Then, the standard curve between the DNA content and copy number of raccoon meat was established.

#### 2.3.6 Construction of an adulteration model

An artificial adulterated model of raccoon meat and pork was established to verify the accuracy of conversion formula between raccoon mass and copy number. Genomic DNA of meat samples with known adulteration ratios was extracted (the ratios of raccoon meat to pork were at 1:9, 2:8, 3:7, 4:6, 5:5, 6:4, 7:3, 8:2, and 9:1, respectively, and the total meat mass is 100 mg). After the extraction, the DNA concentration was measured by the Nanodrop 2000 and the DNA was diluted 30 times. Then, 4 μL of extracted DNA was used to perform the reaction, and experiments were repeated three times. The measured value of raccoon weight calculated by the formula should be compared with the real amount of raccoon to evaluate the accuracy of this formula.

#### 2.3.7 Real sample detection

To further verify the accuracy of the method, all sausage product samples (50 different brands of sausage) were purchased from the market, including 15 chicken sausage products, 15 beef sausage products, 10 pork sausage products, and 10 donkey sausage products. All samples were analyzed by ddPCR three times according to the method of Section 2.3.3, and the adulteration ratio was calculated.

### 2.4 Statistical analysis

All statistical analyses were performed using SPSS 17.0 and Excel.

## 3 RESULTS

### 3.1 Specificity validation

**FIGURE 1:**
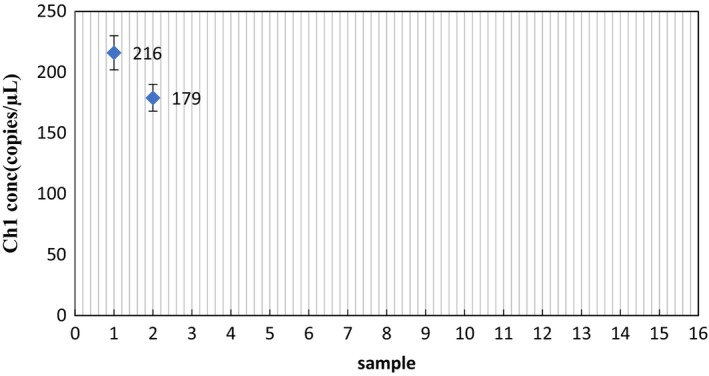
Validation of the raccoon primer probe system. Samples 1–2 raccoon primer template; samples 3–4 raccoon primer–fox template; samples 5–6 raccoon primer–beef template; samples 7–8 raccoon primer–sheep template; samples 9–10 raccoon primer–chicken template; samples 11–12 raccoon primer–pork template; samples 13–14 raccoon primer–duck template; and sample 15 double‐distilled water.

**FIGURE 2:**
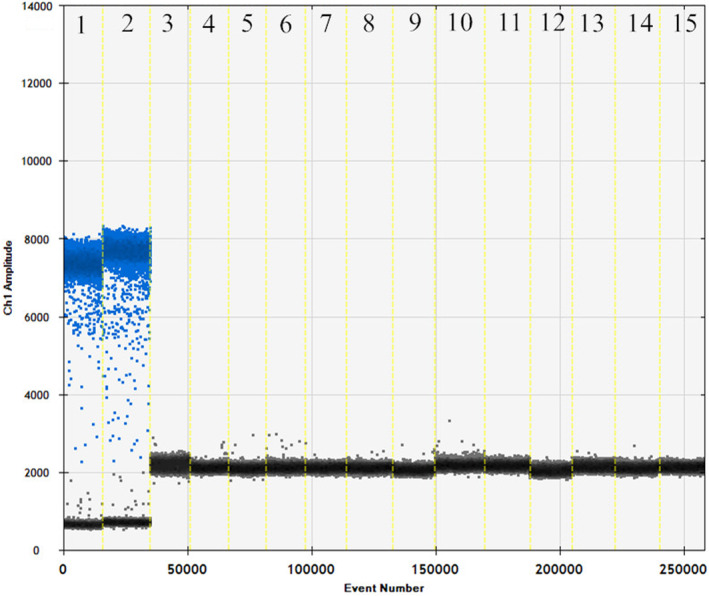
Specificity map of raccoon primer probe. The specificity of raccoon primer probe was tested using the following samples: 1–2 raccoon, 3–4 fox, 5–6 beef, 7–8 sheep, 9–10 chicken, 11–12 pork, 13–14 duck, and 15 negative.

The raccoon‐specific primers and probes were used with raccoon, fox, beef, sheep, chicken, pork, and duck. As shown in Figures 1 and 2, raccoon DNA was used for ddPCR reactions with the specific primers and probes, and the copy number was determined to be 216 and 179 copies/μL. There is no cross‐reaction between specific primers and DNA of other species, which means the primer probe is specific. Thus, it can be used for subsequent detection.

### 3.2 Establishment of the conversion formula between raccoon mass and copy number

#### 3.2.1 Relationship between raccoon weight and extracted DNA concentration

**FIGURE 3:**
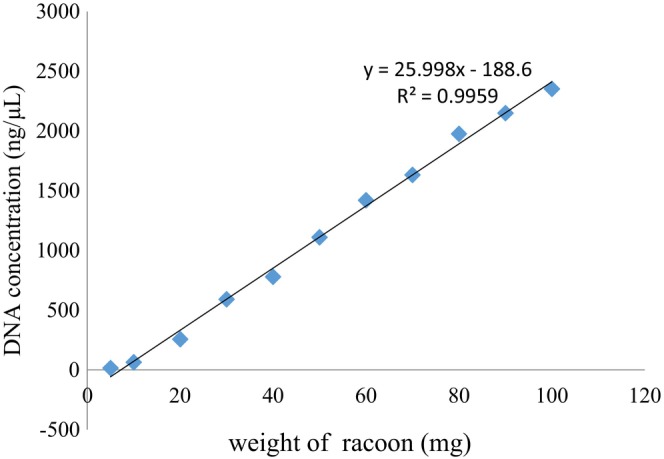
Correlation between racoon mass and extracted DNA concentration.

DNA was extracted from powdered raccoon meat samples ranging from 5 to 100 mg with different mass gradients. DNA concentration was determined using the Nanodrop 2000 Spectrophotometer, and OD260/OD280 ratio was between 1.8 and 2.0. The maximum coefficient of variation of replicates was 7.7%, which is much lower than the specified requirement coefficient of variation of 15% (Table 2). Moreover, there was a linear relationship between DNA concentration and raccoon weight, and the correlation coefficient *R* ^2^ was .9959 (Figure 3). This suggests that the data were stable and reliable.

**TABLE 2:** The raccoon DNA extraction results at different sample masses.

Number	Raccoon mass (mg)	DNA concentration (ng/μL)			Average value (ng/μL)	Coefficient of variation (%)
1	5	15.2	17.1	15.6	16.0	6.3
2	10	59.9	64.5	69.8	64.7	7.7
3	20	254.3	265.6	252.4	257.4	2.8
4	30	587.2	582.5	604.7	591.7	2.0
5	40	802.8	764.1	771.9	779.6	2.6
6	50	1177.1	1031.2	1125.2	1111.2	6.7
7	60	1479.5	1344.7	1437.0	1420.4	4.9
8	70	1709.1	1577.5	1612.3	1633.0	4.2
9	80	2027.1	1955.2	1946.2	1976.2	2.2
10	90	2069.7	2183.3	2198.9	2150.6s	3.3
11	100	2392.1	2303.4	2364.7	2353.4	1.9

#### 3.2.2 Relationship between DNA content and copy number of raccoon meat

**FIGURE 4:**
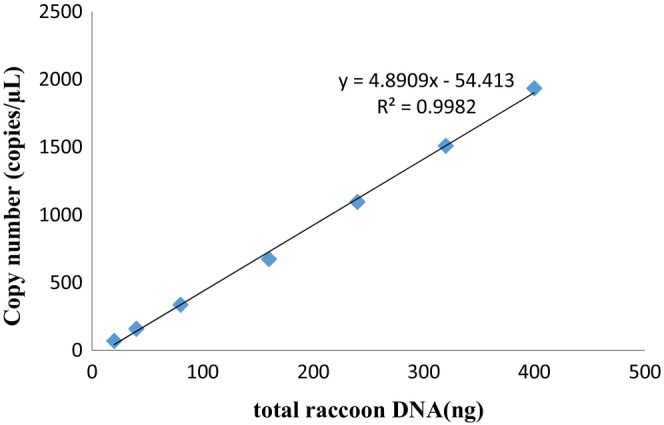
Relationship between raccoon DNA content and copy number.

**FIGURE 5:**
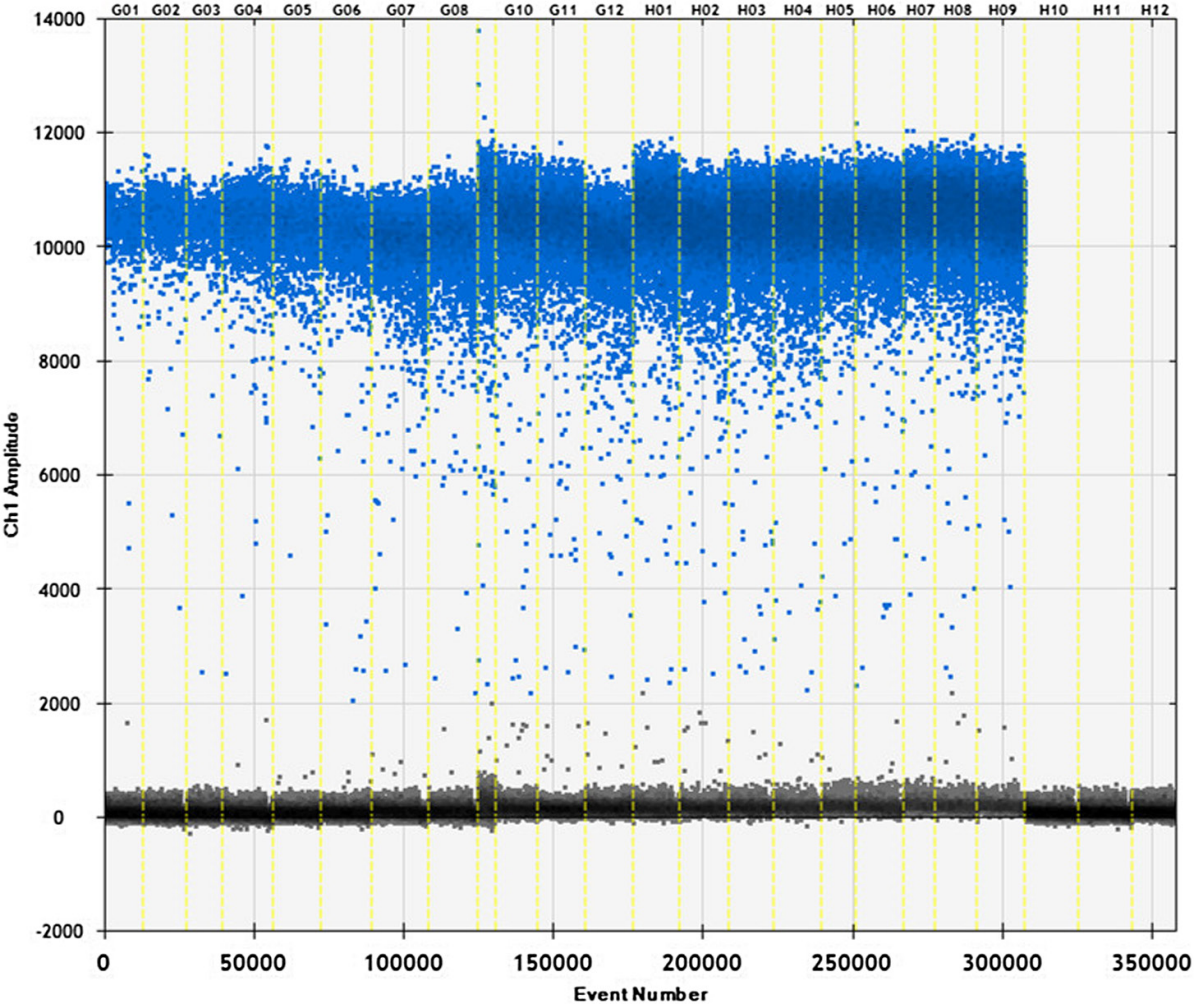
DNA content gradient map of raccoon.

The extracted DNA was diluted to a gradient of 5, 10, 20, 40, 60, 80, and 100 ng/μL and 4 μL of DNA was used to perform the reaction. Figures 4 and 5 show that there was a linear increase in copy number of raccoon meat with increase in DNA concentration, and the correlation coefficient *R* ^2^ was .9982. It can also be seen that the maximum coefficient of variation was 4.6%, which was far below the required coefficient of variation (Table 3).

**TABLE 3:** Raccoon copy number at different DNA contents.

Number	DNA content (ng)	Copy number (copies/μL)			Average value (copies/μL)	Coefficient of variation (%)
1	20	72	71	66	69.7	46
2	40	163	158	159	160.0	1.7
3	80	343	338	329	336.7	2.1
4	160	671	689	663	674.3	2.0
5	240	1101	1097	1091	1096.3	0.5
6	320	1509	1492	1528	1509.7	1.2
7	400	1951	1930	1924	1935.0	0.7

#### 3.2.3 Establishment of the relationship between the weight of raccoon and the copy number of raccoon meat

The adulterated sample with known weight contains unknown adulterated raccoon meat, where *M* is the weight of the unknown adulterated raccoon. The DNA of the samples was extracted using DNA extraction kit and the DNA concentration was measured by the Nanodrop2000. Then, the DNA concentration was diluted 30 times and 4 μL of extracted DNA was used to perform the ddPCR reaction. Therefore, the DNA copy number of the unknown adulterated raccoon can be obtained.

A linear relationship between the weight of raccoon (*M*) and the concentration of extracted DNA concentration (*C*_DNA_) was demonstrated (Figure 3). Meanwhile, the copy number of raccoon meat (*C*) had a linear relationship with extracted DNA content (*T*_DNA_; Figure 4). Furthermore, the DNA concentration was diluted 30 times and 4 μL of extracted DNA was used in ddPCR, thus using the following formula: *T*_DNA_ = *C*_DNA_*4/30. According to these three linear relationships, the formula of raw meat weight (*M*) and the DNA copy number (*C*) was obtained using DNA concentration as the intermediate conversion value (Table 4). The formula is *M* = (*C* + 177.403)/16.954, where *M* is the mass of the raccoon (mg) and *C* is the amplified DNA copy number (copies/μL).

**TABLE 4:** Establishment of raccoon dose–response curve.

Linear curve formula	*R* ^2^
*C*_DNA_ = 25.998 *M* − 188.6	*R* ^2^ = .9959
*C* = 4.8909*T*_DNA_ − 54.413	*R* ^2^ = .9982
*T*_DNA_ = *C*_DNA_*4/30	
*M* = (C + 177.403)/16.954	

Abbreviations: *C*, Copy numbers; *C*_DNA_, DNA concentration; *M*, raccoon mass; *T*_DNA_, total raccoon DNA content.

### 3.3 Method validation–proportionally adulterated model detection

**FIGURE 6:**
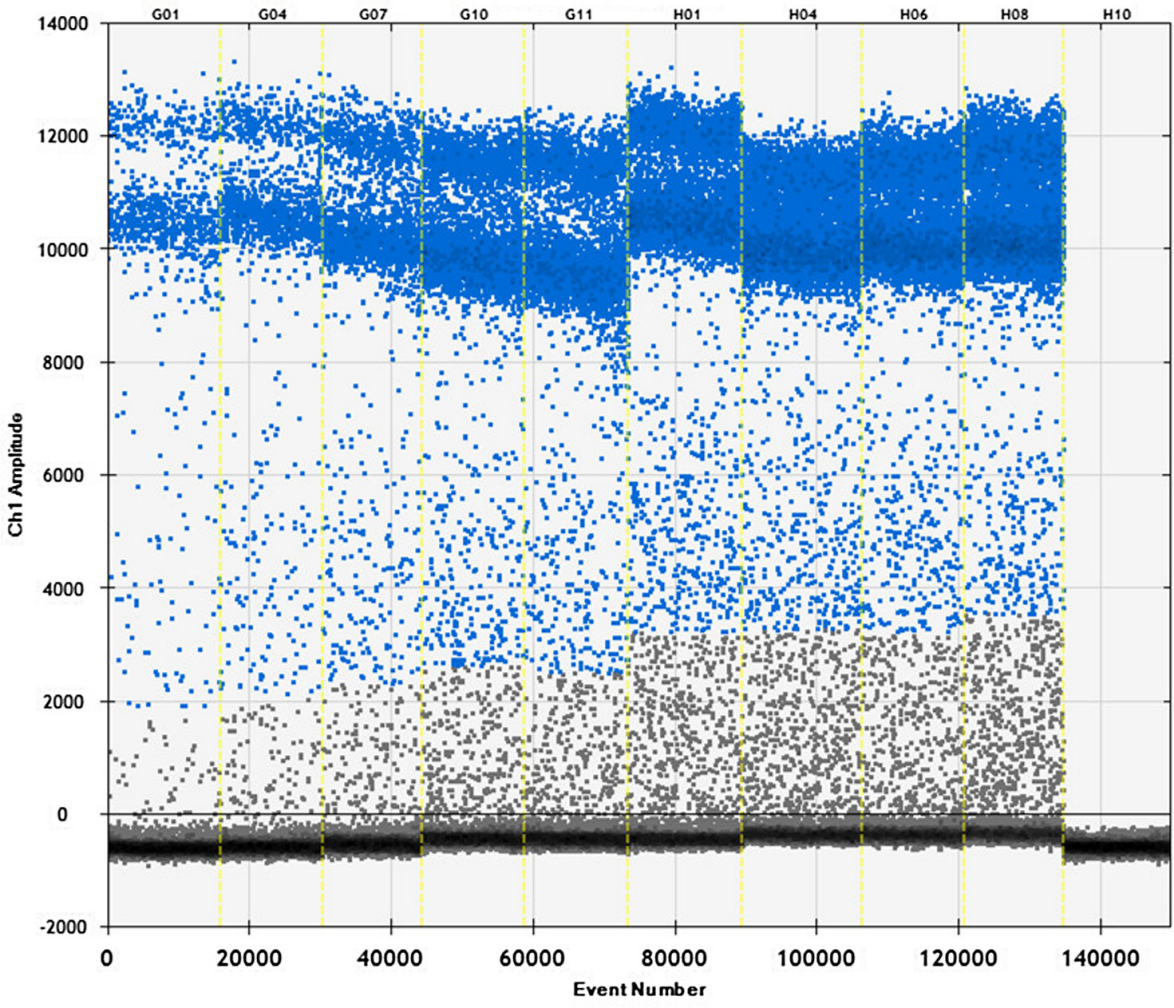
Map of adulterated raccoon.

Proportionally adulterated model detection was used to validate the accuracy of the established ddPCR method. DNA was extracted from 100 mg of mixed raccoon meat, and 4 μL was taken for the experiment. The mass of raccoon meat was calculated by substituting the experimental copy number into the established formula. From Table 5 and Figure 6, the amplification results present that comparing the predicted raccoon meat mass and real mass, the maximum relative error value was 3.7%, which is much lower than the relative error required by the regulations. The amplification results showed that the maximum coefficient of variation of replicates was 2%. This indicates that the established ddPCR method displayed good accuracy, and could be further used for quantitative detection of adulterated raccoon meat in commercial sausage products.

**TABLE 5:** Results of raccoon with known adulterants ratio.

Number	Raccoon mass (mg)	DNA copy number (copies/μL)			Average value (copies/μL)	Coefficient of variation (%)	Calculated raccoon mass (mg)	Relative error (%)
1	20	152	149	146	149.0	2.0	19.3	−3.7
2	30	324	315	312	317.0	2.0	29.2	−2.8
3	40	481	496	484	487.0	1.6	39.2	−2.0
4	50	682	688	674	681.3	1.0	50.7	1.3
5	60	852	859	867	859.3	0.9	61.1	1.9
6	70	1047	1021	1041	1036.3	1.3	71.6	2.3
7	80	1196	1174	1190	1186.7	1.0	80.5	0.6
8	90	1367	1364	1379	1370.0	0.6	91.3	1.4
9	100	1501	1497	1514	1504.0	0.6	99.2	−0.8

### 3.4 Detection of commercially available samples

Fifty different brands of commercially available meat sausages were analyzed using the established ddPCR method. The total weight of the meat sausage was 100 mg, and 4 μL of extracted DNA was used to perform the reaction. It follows from Table 4 that the formula of raccoon meat weight (*M*) and DNA copy number (*C*) is *M* = (*C* + 177.403)/16.954, therefore the calculation formula for the adulteration rate was as follows: adulteration rate (%) = *M*/100*100%. The experiment was repeated three times and the mean values were calculated (Table 6). One of the fifteen chicken sausage products had raccoon adulteration with an adulteration ratio of 15.3%. The highest adulterated raccoon meat was 58.7% in beef sausage products, and five of the 15 beef sausages had raccoon adulterated meat. No raccoon adulteration was tested in 10 pork sausage products. The highest measured raccoon adulteration in donkey meat sausages was 54.1%, and four of the 10 donkey meat sausages had raccoon adulteration. This indicates that raccoon adulteration can be quantitatively identified.

**TABLE 6:** Adulteration mass ratio of commercially available samples.

Sample name	Number	Copy number (copies/μL)			Average value (copies/μL)	Adulteration mass ratio (%)
Chicken sausage	1	0	0	0	0	0
	2	0	0	0	0	0
	3	0	0	0	0	0
	4	0	0	0	0	0
	5	0	0	0	0	0
	6	0	0	0	0	0
	7	0	0	0	0	0
	8	0	0	0	0	0
	9	0	0	0	0	0
	10	0	0	0	0	0
	11	0	0	0	0	0
	12	72	96	76	81.3	15.3
	13	0	0	0	0	0
	14	0	0	0	0	0
	15	0	0	0	0	0
Beef sausage	1	21	12	16	16.3	11.4
	2	0	0	0	0	0
	3	0	0	0	0	0
	4	424	458	437	439.7	36.4
	5	0	0	0	0	0
	6	0	0	0	0	0
	7	0	0	0	0	0
	8	0	0	0	0	0
	9	0	0	0	0	0
	10	28	46	31	35.0	12.5
	11	0	0	0	0	0
	12	836	804	814	818.0	58.7
	13	0	0	0	0	0
	14	212	184	205	200.3	22.3
	15	0	0	0	0	0
Pork sausage	1	0	0	0	0	0
	2	0	0	0	0	0
	3	0	0	0	0	0
	4	0	0	0	0	0
	5	0	0	0	0	0
	6	0	0	0	0	0
	7	0	0	0	0	0
	8	0	0	0	0	0
	9	0	0	0	0	0
	10	0	0	0	0	0
Donkey meat sausage	1	424	411	416	417.0	35.1
	2	0	0	0	0	0
	3	0	0	0	0	0
	4	0	0	0	0	0
	5	51	37	40	42.7	13.0
	6	0	0	0	0	0
	7	725	751	744	740.0	54.1
	8	406	411	387	401.3	34.1
	9	0	0	0	0	0
	10	0	0	0	0	0

## 4 DISCUSSION

Analytical techniques for methods targeting proteins and DNA molecules have been proposed for meat adulteration determination, such as nucleic acid detection technologies, mass spectrometry, and spectrometric techniques. Pan et al. (2020) used DNA barcode technology combined with NGS to accurately identify the problem of various types of meat adulteration and even showed good detection performance in samples mixed with multiple types of meat and reprocessed samples. A real‐time loop‐mediated isothermal amplification method for detecting pig genes in meat products has been established by Cai et al., which is the first attempt to use real‐time LAMP to detect pig‐derived components in commercial products (Cai et al., 2020). Furthermore, Tian et al. (2019) conducted an odor fingerprint investigation on lamb meat adulterated with pork using an electronic nose system consisting of 10 selective MOS gas sensors using PEN 2 (Airsense, Germany). However, the initial modeling and subsequent data processing involved in spectral analysis technology are arduous and unsuitable for efficient on‐site detection. The preprocessing of mass spectrometry samples is time consuming, costly, and necessitates the expertise of skilled personnel. PCR and its associated technologies, renowned for their remarkable efficiency and sensitivity, have emerged as the predominant method for identifying instances of meat adulteration.

PCR technology offers a high potential for the detection of meat adulteration, but it mainly includes the adulteration of common ingredients of livestock and poultry (Cai et al., 2014; Song et al., 2014). So far, only a few studies deal with the detection of non‐edible meat ingredient adulteration and the current meat adulteration by ordinary PCR or fluorescent PCR can only be studied from a qualitative perspective. It is impossible to distinguish between deliberate adulteration of meat products and unconscious contamination during the process of processing and production, which brings loopholes to the current market supervision. The quantitative detection technology can accurately and quantitatively measure the amount of adulterants. In general, the adulteration rate is less than 5% for unintentional adulteration and higher than 10% for intentional adulteration (Chen, Zhang, et al., 2020; Cheng et al., 2020). Therefore, the quantitative study of meat‐derived components and the solution to the supervision and control of meat adulteration in the market have become the focus of current research. Of these, Chen, Chen, et al. (2020) used digital PCR to conduct quantitative detection of duck adulteration in beef and beef meat products. Temisak et al. (2020) employed a triple ddPCR technique to detect pork, beef, and chicken concurrently, utilizing the actin gene and muscle growth inhibitor gene as markers. The developed method exhibited a high sensitivity, with a detection limit of 0.01%. However, the current body of research pertaining to the use of ddPCR for detecting meat adulteration primarily concentrates on commonly consumed meats, with no exploration of its application in identifying adulteration in non‐edible meats.

In this study, we used lean raccoon meat as an experimental model sample, primers and probes were designed based on sequences belonging to single‐copy genes, and DNA concentration was used as the median signal to establish the standard linear relationship between raw raccoon meat mass and copy number. Then, the weight of raccoon meat was calculated directly from the copy number, and *R* ^2^ was all above .99. The method has strong specificity, good repeatability, and no cross‐reaction with other species. Besides, it has a good detection effect on both fresh and cooked meat.

To verify the accuracy of the established ddPCR adulteration method, the adulteration detection experiment was carried out by simulating the adulteration model of the raccoon with a certain mass. The results showed that the maximum weight ratio of the experimental raccoon to the actual added raccoon was 3.7% and the minimum weight ratio was 0.6%. In addition, the practical application value of the method is proved through the actual detection of the market products. Therefore, this method could be applied to detect various sausage products and perform the quantitative identification of animal‐derived components of raccoons.

## 5 CONCLUSIONS

There are various types of sausage products on the market at present. However, adding various additives to meat sausages increases the difficulty of identifying non‐edible meat ingredients. In addition, there is no mature standard detection method for particular meat sources. In this paper, the actual application detection method of the raccoon meat adulteration detection system was established by ddPCR. On the one hand, the establishment of this method fills the gap in the detection of non‐edible meat source adulteration. On the other hand, it can distinguish whether meat products are intentionally adulterated or unconsciously contaminated during the processing and production process. The method provides a scientific basis for the quantitative detection of various kinds of adulterated meat sausage products and also provides a reliable technical means and law enforcement basis for the identification and detection of raccoon meat adulteration.

## AUTHOR CONTRIBUTIONS

**Hui Wang:** Writing – original draft (equal). **Chen Chen:** Validation (equal). **Mengying Xie:** Writing – review and editing (supporting). **Yan Zhang:** Supervision (equal). **Boxu Chen:** Software (equal). **Yongyan Li:** Resources (equal); validation (equal). **Wenshen Jia:** Resources (equal). **Jia Chen:** Resources (equal). **Wei Zhou:** Writing – review and editing (equal).

## FUNDING INFORMATION

This work was funded by the Key Research and Development Program of Hebei Province, China (grant number 21375501D), and the scientific research program of Hebei market supervision administration: study on quantitative identification of particular animal‐derived components in meat and meat products (2023ZD17).

## CONFLICT OF INTEREST STATEMENT

The authors declare that they have no conflicts of interest regarding the publication of this paper.

## Data Availability

The data used to support the findings of this study are included in the article.
